# How Canada can help global adolescent health mature

**DOI:** 10.1186/s12978-017-0353-y

**Published:** 2017-08-10

**Authors:** Ashley Vandermorris, Zulfiqar A Bhutta

**Affiliations:** 1 SickKids Centre for Global Child Health, Toronto, Canada; Division of Adolescent Medicine, Department of Paediatrics, The Hospital for Sick Children and The University of Toronto, Toronto, Canada; 2 SickKids Centre for Global Child Health, Toronto, Canada; SickKids Research Institute, Toronto, Canada; Department of Pediatrics, Department of Nutritional Sciences, and the Dalla Lana School of Public Health, The University of Toronto, Toronto, Canada; Center of Excellence in Women and Child Health, Aga Khan University, Karachi, Pakistan

**Keywords:** Health policy, Adolescent health, Global health

## Abstract

**Background:**

There is an emerging focus on adolescent health within the global health community as we come to recognize that the adolescent years are formative in determining health and health-related behaviours across the life-course. Such attention is not only relevant on the global scale but is imperative in Canada as well.

**Main body:**

This commentary provides a brief review of recent investments targeting global adolescent health and presents five potential avenues for action which emerged out of the recent Canadian Partnership for Women and Children’s Health (CanWaCH) Global Adolescent Health conference. These avenues are: (1) Demand data; (2) Embrace complexity; (3) Be holistic; (4) Engage adolescents; and (5) Commit to Canada.

**Conclusion:**

As international agencies signal their commitment to global adolescent health, Canada is well-positioned to lead this call to action by espousing the fundamental adolescent health tenets of advocacy, equity, justice, and collaboration in order to move this critical agenda forward.

Global adolescent health is coming of age. After being historically overlooked by the health and development communities, the world’s 1.2 billion adolescents are now being recognized as a critical demographic full of promise and potential [1]. As almost 90% of these adolescents live in low- and middle-income countries (LMICs), engaging this group successfully demands an integrated international effort that recognizes the varied challenges and contexts in which adolescents live.

Many groups have recently signaled their commitment to enhancing global adolescent health, acknowledging that investments in adolescents can yield what a 2016 Lancet Commission termed the “triple dividend”: improvements in adolescents’ current health, future health, and the health of their future offspring [2]. The Global Strategy for Women’s, Children’s, and Adolescents’ Health 2016–2030, led by the UN Secretary General and the World Health Organization (WHO), includes over $25 billion in dedicated funding and explicitly acknowledges that addressing adolescent health will be critical to achieve the Sustainable Development Goals [3]. In March of this year, Canada took on a leadership role in promoting adolescent health by pledging $650 million over three years to support global initiatives to address the sexual and reproductive health and rights of women and girls [4].

What does this all mean? How can Canada ensure that the field of global adolescent health matures in a manner that is responsible, productive, resilient, robust and principled – and in so doing ensure that the field supports adolescents throughout the world in achieving a similarly healthy and positive development?

In May of this year, leaders in global adolescent health convened in Ottawa for the Canadian Partnership for Women and Children’s Health (CanWaCH) Global Adolescent Health conference and the launch of the WHO’s guidance document: Global Accelerated Action for the Health of Adolescents (AA-HA!) [5]. The meeting brought together key stakeholders—including, importantly, a globally representative youth council—and afforded an opportunity to share in successes, reflect on challenges and, most critically, to discuss a coordinated, comprehensive path forward. Out of these discussions emerged five themes that can position Canada to successfully champion global adolescent health.

## Demand data

It is a common misconception that we don’t yet have an adequate research base to take effective action on adolescent health. In fact, a robust body of evidence has been generated through recent systematic reviews of health and nutrition interventions, and has been made easily accessible to policy-makers and programmers through the AA-HA! guidance package of evidence-based interventions. We know what should work. What now requires priority on the research agenda are the questions of what works in what contexts, for whom, and, ideally, why. To understand these questions, we must take a two-pronged approach: we must promote and invest in rigourous, scientifically-sound evaluations of existing interventions, and we must lead the charge in refocusing our research endeavours towards implementation studies. Such a shift in the orientation of research, coupled with sustained pressure to disaggregate all health data by age and sex and to strengthen health management and information systems, will allow for a more nuanced, comprehensive, and ultimately applicable foundation of knowledge on which to base next steps.

## Embrace complexity

Adolescents are complex, and working with them must embrace this complexity rather than take a reductionist approach. This time of significant physical, emotional, and cognitive development is the stage within the life course with perhaps the most varied and culturally-specific set of socially-embedded expectations, beliefs and perceptions. Successful programming for adolescents not only takes such complexity into account but leverages it towards positive outcomes. Priority-setting must be consultative and recognize the interdependencies within the networks in which adolescents exist. Transformation must be allowed to emerge organically and then scaffolded with well-defined criteria that direct change towards meaningful outcomes. Relationships must be cultivated and nurtured: recognizing that adolescent health is shaped largely by determinants that fall outside of the traditional health sector, actions targeting adolescent health will require integrated intersectoral collaboration and coordination.

## Be holistic

Historically, programming for adolescents has almost exclusively targeted their sexual and reproductive health. The AA-HA! guidance document challenges this legacy and promotes a whole-person view of adolescents. An adolescent’s forced early marriage will have negative implications for her health, her ability to continue her education, and her future career opportunities. A teenager’s untreated depression will put him at risk of exposure to violence, substance use, chronic health conditions, and poorer educational outcomes. Adolescents must have access to a comprehensive package of evidence-based interventions that promote health holistically through targeting positive development, unintentional injury, violence, sexual and reproductive health, communicable disease, non-communicable diseases, nutrition, physical activity, mental health, substance abuse and self-harm [5]. Developing and delivering such interventions must also be holistic. Public policy should be adolescent-responsive and informed by what recent advances in neuroscience have revealed about the adolescent brain. Adolescent Health in All Policies, defined as “an approach to public policies across sectors that systematically takes into account the implications of decisions for adolescent health, avoids harmful effects and seeks synergies – in order to improve adolescent health and health equity”, is one means of achieving this critical goal [5]. Furthermore, it must also be understood that many interventions with the potential to impact adolescent health and nutrition outcomes must be initiated within the school age population for optimal effect, hence underscoring the need for better integration of strategies across the life course.

## Engage adolescents

Adolescents have unique needs, aspirations, ambitions and abilities. Simply expanding existing programs to include adolescents may meet certain short-term needs but overlooks the incredible opportunity for health system transformation that could emerge from the meaningful engagement of youth. The AA-HA! guidance document suggests that programming must be “WITH adolescents, FOR adolescents” [5]. Adolescents’ position in society means that their successful engagement will depend on also engaging their families and communities, with the shared goal of supporting adolescents’ emerging autonomy. Sustainable engagement and inclusion of adolescents and adolescent voices in the development and implementation of adolescent health initiatives is critical to ensure operational success and crucial from an ethical and human rights perspective.

## Commit to Canada

Ninety per cent of the world’s adolescents may live in LMICs, but over 3.9 million 10–19 year olds live in Canada, and drastic disparities in their health outcomes persist [6]. Suicide rates among First Nations youth are 5–7 times higher than non-Aboriginal youth; at eleven times the national average, suicide rates among Inuit youth are among the highest in the world [7]. Indigenous adolescents living in Canada continue to face significant barriers to accessing culturally-relevant and respectful mental health, physical health, educational and employment services. The designation of a Minister of Youth, held by the Prime Minister himself, the development of the Prime Minister’s Youth Council, and the attendance of the Parliamentary Secretary to the Prime Minister (Youth) at the recent CanWaCH Global Adolescent Health conference all signal a recognition of the need to make adolescents more prominent on the domestic agenda. Canada has the privilege of resources and infrastructure that many LMICs do not. It is imperative that we model leadership by collaborating with, investing in, researching for, and learning from Canadian adolescents of varied backgrounds, geographies, and identities to support the positive trajectories of our own adolescents, both for the sake of our own current and future society, and if we wish to have a legitimate role in leading the call for accelerated action on adolescent health around the world.

## Conclusions

Canada is well-positioned to lead the global health community in advancing efforts to improve global adolescent health. Effective leadership will emerge from espousing the fundamental adolescent health tenets of advocacy, equity, justice, and collaboration.

## Abbreviations

AA-HA!: Global Accelerated Action for the Health of Adolescents
CanWaCH: Canadian Partnership for Women and Children’s Health
LMICs: low- and middle-income countries
WHO: World Health Organization
